# Mechanisms of CFTR Folding at the Endoplasmic Reticulum

**DOI:** 10.3389/fphar.2012.00201

**Published:** 2012-12-13

**Authors:** Soo Jung Kim, William R. Skach

**Affiliations:** ^1^Department of Biochemistry and Molecular Biology, Oregon Health and Science UniversityPortland, OR, USA

**Keywords:** cystic fibrosis, CFTR, membrane protein biogenesis, protein translocation, cotranslational folding, nucleotide binding domain, ABC transporter

## Abstract

In the past decade much has been learned about how Cystic Fibrosis Transmembrane Conductance Regulator (CFTR) folds and misfolds as the etiologic cause of cystic fibrosis (CF). CFTR folding is complex and hierarchical, takes place in multiple cellular compartments and physical environments, and involves several large networks of folding machineries. Insertion of transmembrane (TM) segments into the endoplasmic reticulum (ER) membrane and tertiary folding of cytosolic domains begin cotranslationally as the nascent polypeptide emerges from the ribosome, whereas posttranslational folding establishes critical domain–domain contacts needed to form a physiologically stable structure. Within the membrane, N- and C-terminal TM helices are sorted into bundles that project from the cytosol to form docking sites for nucleotide binding domains, NBD1 and NBD2, which in turn form a sandwich dimer for ATP binding. While tertiary folding is required for domain assembly, proper domain assembly also reciprocally affects folding of individual domains analogous to a jig-saw puzzle wherein the structure of each interlocking piece influences its neighbors. Superimposed on this process is an elaborate proteostatic network of cellular chaperones and folding machineries that facilitate the timing and coordination of specific folding steps in and across the ER membrane. While the details of this process require further refinement, we finally have a useful framework to understand key folding defect(s) caused by ΔF508 that provides a molecular target(s) for the next generation of CFTR small molecule correctors aimed at the specific defect present in the majority of CF patients.

## Introduction

Cystic fibrosis (CF) is one of a growing number of human diseases caused by inherited mutations that disrupt protein folding. It is caused by dysfunction of the Cystic Fibrosis Transmembrane conductance Regulator (CFTR), a cAMP-regulated ion channel that resides in the apical membrane of epithelial cells (Riordan, 2008; Lubamba et al., 2012). CFTR dysfunction can occur by defects in protein synthesis, folding, intracellular trafficking, channel gating, chloride conductance, or plasma membrane stability. In each case, loss of CFTR results in abnormalities of water, chloride, and/or bicarbonate transport that lead to dysfunction of target tissues including: pancreatic insufficiency, increased sweat chloride, intestinal obstruction, and most importantly, chronic pulmonary infection, inflammation, and ultimately death due to respiratory failure (Cohen and Prince, 2012; Ratjen and McColley, 2012). The most prevalent CFTR mutation, Phe508del (ΔF508), is found in ∼90% of CF patients (Riordan et al., 1989) where it impairs CFTR folding, inhibits channel gating, and decreases plasma membrane stability (Lukacs and Verkman, 2012). The mechanisms by which ΔF508 disrupts CFTR folding are beginning to be understood, and small molecule modulators that restore endoplasmic reticulum (ER) trafficking and channel gating hold great promise for new treatments to correct these underlying molecular abnormalities in CF patients.

**Figure 1:**
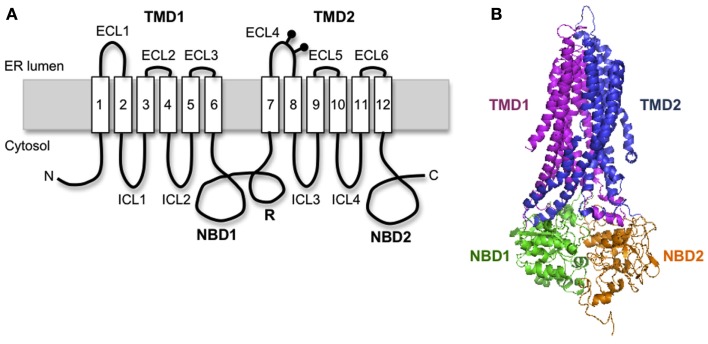
**Cystic fibrosis transmembrane conductance regulator structural organization**. **(A)** Schematic diagram of CFTR showing transmembrane topology and domain organization. **(B)** A predicted human CFTR structure based on homology model from Sav1866.

Cystic fibrosis transmembrane conductance regulator is a 1480 amino acid polytopic glycoprotein in the ABC transporter family (ABCC7) that contains two six-spanning transmembrane (TM) domains (TMD1 and TMD2) that form the channel pore, two cytosolic nucleotide binding domains (NBD1 and NBD2) that drive channel gating, and an intrinsically unstructured regulatory (R) domain that controls channel activity via PKA-mediated phosphorylation (Figure 1A). CFTR synthesis has been estimated to take 9–10 min in eukaryotic cells (Ward and Kopito, 1994), suggesting that significant folding occurs cotranslationally. Like most polytopic membrane proteins, CFTR biogenesis occurs at the ER, and requires coordinated folding of individual domains in three distinct cellular compartments: the ER membrane, the ER lumen, and the cytosol. This compartmentalization takes place as the nascent chain emerges from the ribosome. Subsequent assembly of TMDs and NBDs into the final folded structure takes ∼30–120 min and is facilitated by a large cohort of cytosolic and lumenal chaperones including Hsp70, Hsp40, Hsp90, calnexin, and others (Amaral, 2004; Skach, 2006; Wang et al., 2006). If CFTR fails to achieve its native fold, chaperones such as Hsp70 also act to recruit E3 (and/or E4) ubiquitin-ligases that ubiquitinate CFTR and target the mutant protein for degradation by the 26S proteasome. Thus, CFTR folding is constantly monitored by cellular quality control machinery throughout its biogenesis.

This review will focus on the current state of knowledge as to how CFTR domains fold, how they interact, how mutations alter this process, and how misfolded conformations are distinguished from native structure by cellular chaperone machinery.

## Multispanning Membrane Protein Biogenesis at the ER

**Figure 2:**
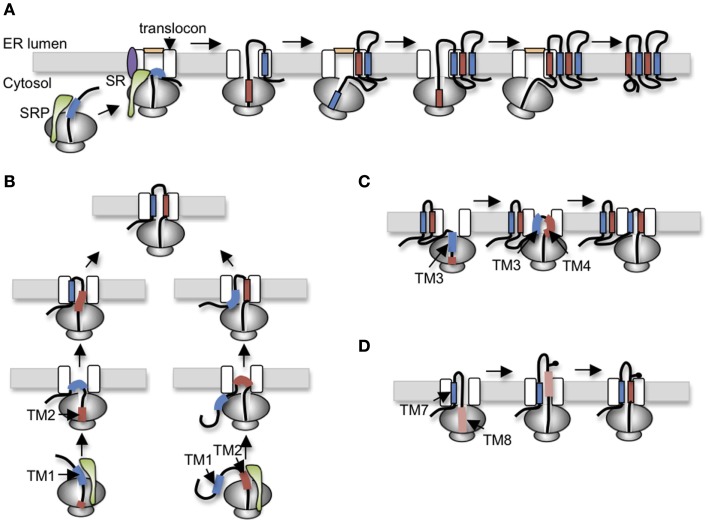
**Variations of polytopic protein topogenesis**. **(A)** Simplest cotranslational topogenesis model in which ER targeting begins as signal recognition particle (SRP) recognizes an emerging signal sequence (TM), binds its receptor (SR) at the ER membrane, and transfers the RNC to the Sec61 translocon. TM topology is achieved through alternating signal and stop transfer activities that sequentially open and close the translocon pore. Careful orchestration of ribosome translocon junction ensures delivery of soluble domains into either cytosol or ER lumen and integration of TMs into the bilayer. **(B)** For CFTR, topology of TM1 and TM2 is established by two alternate pathways in which translocation is initiated by either TM1 (left) or TM2 (right). Most CFTR nascent chains utilized a posttranslational mechanism in which TM2 insertion drags TM1 into the translocon. **(C)** The short loop between TM3 and TM4 (five residues) suggests that TM3 and TM4 simultaneously insert into the translocon as a helical hairpin. A similar mechanism is also proposed for TM5-6, TM9-10, and TM11-12. **(D)** Stop transfer activity of TM8 is weakened by Asp924 which results in transient exposure of TM8 in the ER lumen before acquiring its final membrane spanning topology.

To understand specialized aspects of CFTR biogenesis, it is helpful to first consider general mechanisms. In eukaryotic cells, membrane proteins are targeted to the ER during synthesis by the cytosolic signal recognition particle (SRP; Walter and Blobel, 1981), which brings the ribosome nascent chain complex (RNC) to the Sec61 translocon (Figure 2A). As the RNC docks onto the translocon, the insertion of the signal sequence into Sec61α opens the protein conducting channel (PCC) and establishes a continuous aqueous pathway from the ribosome exit tunnel into the ER lumen (Figure 2; Crowley et al., 1993, 1994). Extracellular peptide loops generally pass through the PCC cotranslationally until synthesis of a hydrophobic TM segment (i.e., stop transfer sequence) terminates nascent chain translocation (Haigh and Johnson, 2002; Woolhead et al., 2004; Alder et al., 2005) and relaxes the ribosome translocon junction to allow the downstream peptide region access to the cytosol (Liao et al., 1997). TM segments also move laterally out of the translocon as they integrate into the lipid bilayer. In some cases, integration occurs via a passive thermodynamic partitioning (Martoglio et al., 1995; Heinrich et al., 2000), whereas in others, it appears to be mechanistically controlled by the ribosome translocon complex (RTC; Do et al., 1996; Pitonzo et al., 2009). Indeed, TMs may be released from the translocon individually, in pairs, or even groups depending on specific properties and folding requirements of the substrate (Meacock et al., 2002; McCormick et al., 2003; Sadlish et al., 2005). Crystal structures of the Sec61αβγ homolog from *M. jannaschii* (SecYEβ) have suggested that TMs exit the translocon via a lateral cleft between Sec61α TMs2-3 and TMs7-8 along one side of the PCC (Van den Berg et al., 2004). Functional mammalian translocons also contain additional translocon-associated proteins including the translocation-associated membrane protein (TRAM), translocon-associated membrane protein (TRAP) complex, signal peptidase complex, oligosaccharyltransferase (OST), and others that modulate translocation, integration, and early processing events (Schröder et al., 1999; Wang and Dobberstein, 1999; Shibatani et al., 2005). Thus, the Sec61αβγ PCC functions as part of a large integrated molecular machine.

In the simplest model, polytopic protein topology could be established by alternating TMs (encoding signal or stop transfer activity) that sequentially open the translocon pore into the ER lumen to initiate translocation and close the pore to terminate translocation and direct peptide segments into the cytosol. Such a mechanism would maintain ER integrity while essentially stitching TM segments into the bilayer via coordinated structural changes at the lumenal and the cytosolic faces of the RTC (Johnson, 2003; Sadlish and Skach, 2004; Pitonzo and Skach, 2006; Skach, 2009).

## CFTR Folding

### CFTR TM insertion and TMD formation

Homology models predict that CFTR exhibits a complex domain swap structure in which two six-spanning helical bundles containing TMs1-2, 9-12 and TMs7-8, 3-6 are twisted around a central ion-conducting pore (Locher et al., 2002; Dawson and Locher, 2006; Aller et al., 2009). Helical extensions of the TMs form intracellular loops (ICL1-4) that project nearly 40 Å into the cytosol and form docking sites for NBD1 and NBD2 (Figure 1B). It is currently believed that ATP binding and hydrolysis at the interface between the two NBDs transmits an allosteric conformational change along the ICLs to the TMDs that controls channel gating. This elegant structure immediately raises several important questions when considered from a biosynthetic viewpoint. First, how do CFTR TMs acquire their proper topology as they are oriented and integrated into the ER membrane? Second, how do TMs interact during TMD assembly? Third, where do domain swapping and assembly occur in relation to the translocon, i.e., where do TMs1-2 and TMs3-6 transiently reside for the several minutes that it takes to synthesize TMs7-8 and TMs9-12? An important consideration is that CFTR TMs contain an unusually large number of potentially ionizable residues (4 Arg, 2 Lys, 3 Glu, 1 Asp, and 1 His), which likely establish a network of polar interactions within the membrane. However, such residues would be predicted to delay or destabilize integration of individual TMs in the bilayer (Hessa et al., 2005). In addition, mutagenesis studies have revealed that TMD assembly influences folding of cytosolic NBDs and visa versa (Chen et al., 2004; Loo et al., 2008), such that domain folding and domain–domain assembly exhibit a high degree of cooperativity.

One of the first identifiable features of CFTR folding involves the orientation and integration of TMs into the ER membrane. Early work from our group established that ER targeting occurs as TM1 and TM2 emerge from the ribosome, bind SRP (Carlson et al., 2005), and engage the Sec61 translocon via a novel mechanism that involves two alternative folding pathways (Lu et al., 1998). Notably, TM1 lacks efficient signal anchor activity due to the presence of two ionizable residues, Glu92 and Lys95, within its membrane spanning region. As a result, TM1 initiates translocation for only ∼25% of nascent CFTR polypeptides. For the remaining 75% of chains, topology of the TM1-2 loop is established by type I signal anchor activity of TM2. In this case, the energy of TM2 insertion into the translocon, essentially “drags” the first extracellular loop (ECL1) into the ER lumen, thereby establishing the type II topology of TM1 (Figure 2B). While the final outcome of the two pathways is identical, the latter differs from the simple cotranslational model because TM1 acquires its topology after TM2. Such a mechanism suggests that both TMs are accommodated simultaneously either within or closely adjacent to the translocon channel.

An important implication of this topogenesis mechanism is highlighted by two CF-causing mutations, G85E and G91R, each of which introduces an additional ionizable residue into TM1. Both mutants completely block TM1 signal anchor activity but do not affect TMD1 topology because TM1 can still be inserted into the membrane by TM2 (Xiong et al., 1997). Despite achieving correct topology, however, G85E and G91R still disrupt CFTR folding and trafficking (Xiong et al., 1997; Patrick et al., 2011). Analysis of TM1-2 topogenesis gave rise to the early prediction that disease related mutations in different regions of CFTR might disrupt folding via a common mechanism, namely by preventing higher order tertiary domain–domain interactions (Xiong et al., 1997; Skach, 2000). In the case of TM1, we proposed that insertion of an additional polar residue disrupted the arrangement of helical bundles and subsequent interactions between helical extensions and cytosolic NBDs (Xiong et al., 1997; Skach, 2000). This finding led to the early proposal that normal CFTR folding requires precise formation of domain–domain contacts, similar to a molecular jig-saw puzzle, which has recently been shown by several groups to be a major defect in the ΔF508 mutation as well (Serohijos et al., 2008; Mendoza et al., 2012; Rabeh et al., 2012).

TM3 also encodes an inefficient signal sequence that cooperates with TM4 to translocate the intervening extracellular loop, ECL2. Because ECL2 contains only five residues it is likely that TM3 and TM4 insert simultaneously into the translocon pore as a helical hairpin (Figure 2C). Similarly, TM5 and TM6, which function as signal anchor and stop transfer sequences, are separated by only a single charged lysine residue, indicating that their topology is also established together as ECL3 is translocated into the ER lumen. This feature of coincident translocation by TM helical hairpins is a common feature of native polytopic proteins (Sadlish et al., 2005) and suggests that two closely spaced TMs could be considered as a single functional topogenic determinant. However, few studies have investigated the mechanism by which such determinants interact with the translocon to establish topology.

From these results, we propose a general, although admittedly incomplete model in which TM1-2 topology is acquired through the combined actions of weak type II SA (TM1) and strong type I SA (TM2) activities. Subsequently, TM3-4 and TM5-6 insert into Sec61 as helical hairpins to translocate short ECL2 and ECL3 loops. An interesting and currently unresolved question is whether TMs or TM pairs partition freely into the bilayer from the small Sec61αβγ pore, if they remain associated with Sec61 and/or other translocon proteins during subsequent helical packing. This question is particularly relevant in light of the mature domain swap structure where TM1-2 ultimately bundles with TM9-12 and TM3-6 with TM7-8. Given the prevalence of ionizable residues in TMs1, 2, 3, and 6, final assembly likely requires precise alignment of TMs prior to complete integration into the lipid bilayer.

When TM6 terminates translocation NBD1 cotranslationally passes beneath the base of the ribosome into the cytosol. Several features of CFTR suggest that during this process, the ribosome transiently disengages from Sec61 to allow folding of the cytosolic domains. CFTR TMD1 helices are predicted to extend ∼40–50 Å from the membrane, and these extensions appear to provide a preliminary docking site for NBD1 prior to synthesis of TMD2 (Xiong et al., 1997; Kleizen et al., 2005; Du and Lukacs, 2009). The TMD extension plus bound NBD1 would therefore extend nearly 80 Å from the membrane surface, requiring that this region must move away from the ribosome to avoid a major steric clash. It is unknown whether a new translocon is recruited for TMD2 topogenesis, or whether preliminary assembly of the N-terminal half of CFTR occurs within or adjacent to the translocon. Precisely where these early folding events might take place and the factors involved remain important as yet unanswered questions.

### Novel mechanisms of CFTR TMD2 topogenesis and folding

After completion of R-domain synthesis, TMD2 topology is established in a cotranslational manner by alternating signal (TM7, 9, and 11) and stop transfer (TM8, 10, and 12) sequences. As TM7 emerges from the ribosome, it efficiently directs membrane targeting and ECL4 translocation. TM8, which is separated from TM7 by ∼31 residues, terminates translocation, and redirects ICL3 in the cytosol as expected. Interestingly, TM8 functions as an efficient stop transfer only when it is normally paired with TM7, but not in a heterologous context (Carveth et al., 2002; Enquist et al., 2009). This suggests that TM7 either influences TM8 stop transfer activity inside the translocon or alternatively, that TM7 affects recognition of TM8 within the ribosome exit tunnel. To date, this type of cooperativity appears unique to CFTR, although few proteins have been studied at this level of detail. The remaining TM pairs, TM9-10 and TM11-12, each encode signal anchor and stop transfer sequences with short extracellular loops, and it is likely that they insert into the translocon as helical hairpins much like TM3-4 and TM5-6 (Carveth et al., 2002).

TMD2 exhibits several additional unusual folding behaviors. It is well known that N-linked glycosylation sites must be at least 12–14 residues from the lipid bilayer to be accessible to OST (Popov et al., 1997; Nilsson and von Heijne, 2000). The CF mutation T908N, however, creates a glycosylation site that is recognized by OST even though it is only four-residues from the predicted N-terminus of TM8. Given that the precise boundaries of CFTR TMs are not yet known, one possible explanation for these findings is that residues within TM8 that actually span the membrane bilayer may differ from current predictions. Alternatively, if TM8 membrane boundaries are accurately predicted by homology models, then this finding suggests that TM8 transiently extends into the ER lumen during CFTR synthesis and is then repositioned within the membrane during subsequent folding and helical packing (Hammerle et al., 2000; Carveth et al., 2002; Figure 2D). Such behavior could be due to either altered interactions with translocon components that fail to recognize TM8 during synthesis, altered timing of TM8 helix formation, or both. Interestingly, removal of an aspartate residue from TM8 (D924V) prevents transient lumenal exposure and at the same time confers independent stop transfer activity. Although the original observation that TM8 might transiently sample the lumenal environment was unexpected, there is growing appreciation that other weakly hydrophobic TMs in polytopic proteins do indeed undergo repositioning within the membrane, either through interactions with neighboring TMs during tertiary folding, or due to differences in membrane thickness and/or composition that occur at various locations along the secretory pathway (Meindl-Beinker et al., 2006; Hessa et al., 2007; Skach, 2009; Nörholm et al., 2011).

Cystic fibrosis transmembrane conductance regulator also exhibits a distinct mechanism of membrane integration. The first clue came from the observation that after synthesis is completed, CFTR remains transiently bound to a large protein complex with properties similar to the RTC (Oberdorf et al., 2005). Release from this complex into the bilayer requires both cytosol and energy. *In vitro* photocrosslinking experiments further demonstrated that TM8 can maintain stable interactions with Sec61α after cleavage of peptidyl tRNA bond, and that release from the translocon also requires ATP (Pitonzo et al., 2009). Surprisingly, Asp924, which influences TM8 stop transfer activity, is also responsible for retaining TM8 within Sec61, suggesting that polar interactions can rigidly hold a TM within the translocon structure (Pitonzo et al., 2009). These results demonstrate that the translocon has the capacity to regulate the timing of TM integration via specific protein–protein interactions and thereby potentially facilitate early steps of TMD assembly (Do et al., 1996; Liao et al., 1997; Skach, 2009).

In summary, CFTR TMD biogenesis utilizes multiple mechanisms that deviate from a cotranslational topogenesis model including: alternate co- and post-translational translocation pathways (TM1-2), coincident insertion of helical hairpins (TM3-4, TM5-6, TM9-10, and TM11-12), cooperativity for topogenic determinant function (TM7-8), and regulated integration into the ER membrane. Reasons underlying these distinct translocation mechanisms are only beginning to be understood, but evidence suggests that different folding pathways have functional implications. For example, replacement of ionizable residues in TM1 (E92A and K95A) converts TM1 to a strong signal anchor sequence, thus favoring cotranslational topogenesis, but disrupts CFTR function (Lu et al., 1998; Patrick et al., 2011). Similarly, the D924V mutation converts TM8 to a strong strop transfer sequence and facilitates cotranslational membrane integration, but decreases CFTR chloride conductance (our observations). These results are mirrored in the mammalian aquaporin family and suggest that by facilitating different topogenesis mechanisms, eukaryotic translocon machinery has allowed TM segments to accommodate key functional residues that would otherwise disrupt cotranslational membrane insertion (Skach, 2009). An obvious but profound implication is that folding and function are closely intertwined such that structural elements needed for higher order folding ultimately dictate which topogenesis mechanisms prevail.

### CFTR cytoplasmic domain folding and the defect of ΔF508

**Figure 3:**
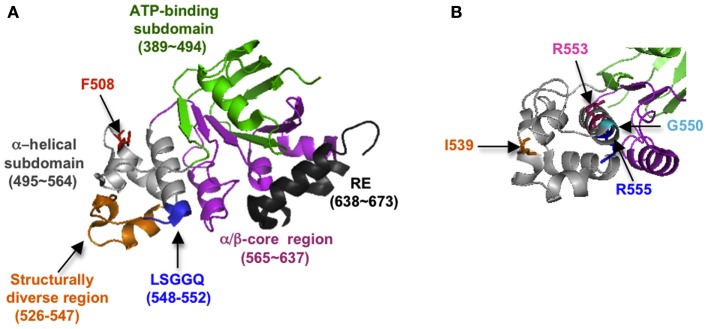
**(A)** Crystal structure of NBD1 showing subdomain organization and location of key structural elements (2BBO). **(B)** Slightly different view of **(A)**, showing location of suppressor mutations (1XMI).

It is now evident that correct folding of individual CFTR domains is required for proper domain assembly, and that proper domain assembly reciprocally influences domain folding (Qu and Thomas, 1996; Younger et al., 2006; Loo et al., 2008; Du and Lukacs, 2009; Thibodeau et al., 2010). Among these processes, NBD1 folding and mechanism(s) by which folding is disrupted by ΔF508 have received intense attention. NBD1 is composed of three subdomains: an N-terminal subdomain that contains the ATP binding site (Khushoo et al., 2011), an α-helical subdomain containing Phe508, and a central α/β core analogous to the F1-type ATPase containing a six-stranded, largely parallel β-sheet (Figure 3). NBD1 also contains the canonical LSGGQ signature motif (residue 548–552), a unique unstructured regulatory insertion (residues 404–436), a structurally diverse region (residues 526–547), and a C-terminal regulatory extension (RE; Figure 3A). Given its profound effect on CFTR folding, it was initially surprising that the ΔF508 mutation has little effect on NBD1 crystal structure (Lewis et al., 2004, 2005). However, recent work has revealed that ΔF508 significantly disrupts both kinetic and thermodynamic stability of NBD1 as well as increasing local backbone dynamics at residues 507–511 (Hoelen et al., 2010; Lewis et al., 2010; Wang et al., 2010; Rabeh et al., 2012). Moreover, the specific folding defect induced by ΔF508 appears to reside at least in part within the α-helical subdomain (Hoelen et al., 2010; Wang et al., 2010) as well as C-terminal β-strands, S9 and S10 (Hudson et al., 2012). ΔF508 also eliminates a hydrophobic contact between NBD1 and TMD2 that is required for trafficking and channel gating (Serohijos et al., 2008).

Mutations that increase NBD1 solubility and/or thermodynamic stability (I539T, G550E, R553Q, and others; Teem et al., 1993; DeCarvalho et al., 2002; Roxo-Rosa et al., 2006; Pissarra et al., 2008; Hoelen et al., 2010) and/or decrease backbone flexibility (Aleksandrov et al., 2012) can enhance both NBD1 folding yield in cells and trafficking efficiency of full length WT as well as ΔF508 CFTR (Figure 3B). Thus NBD1 folding *per se*, is a liming step in both WT and ΔF508 CFTR biogenesis. Mutations within ICL4 or NBD1 that restore NBD1–TMD2 interaction also improve ER export and chloride channel function (Serohijos et al., 2008; He et al., 2010; Loo et al., 2010; Thibodeau et al., 2010; Aleksandrov et al., 2012). However, correction of both the NBD1–TMD2 interface and NBD1 thermodynamic stability are required to restore ΔF508 processing to near wild-type levels (Mendoza et al., 2012; Rabeh et al., 2012). A major goal in CF, therefore, is to identify small molecules that act at both of these folding steps and thereby increase channel function in CF patients.

How then does NBD1 fold in cells, and which limiting steps might provide a target for small molecule correction? NBD1 folding begins cotranslationally after TM6 terminates translocation, and the elongating nascent chain moves into the cytosol through the relaxed ribosome translocon junction (Carveth et al., 2002). It is estimated to take roughly 1 min to synthesize NBD1 in eukaryotic cells, and significant folding (as well as mis-folding of ΔF508) occurs during this time (Kleizen et al., 2005; Hoelen et al., 2010). However, understanding NBD1 cotranslational folding has been technically challenging because of the complex biological folding environment. For example, cotranslational folding is influenced by the rate and vectorial nature of translation (Fedorov and Baldwin, 1997; Siller et al., 2010), the ribosome, and geometry of the ribosome exit tunnel (Woolhead et al., 2004; Lu and Deutsch, 2005; Ziv et al., 2005; Kaiser et al., 2011), molecular crowding (Ellis, 2001), and interaction with several cellular chaperone networks (Frydman, 2001; Ellis, 2007; Hartl and Hayer-Hartl, 2009).

One promising method to define folding transitions as the nascent chain emerges from the ribosome is to measure fluorescence energy transfer (FRET) between Donor and Acceptor probes that are cotranslationally incorporated at distant sites in primary sequence but which become proximal to one another as the protein folds. Because FRET efficiency is highly sensitive to changes in distance on a scale of ∼10–80 Å, changes in FRET that occur at increasing chain lengths provide a sensitive readout for nascent chain compaction and folding. Using this approach, Khushoo et al. (2011) showed that NBD1 folding begins cotranslationally and proceeds via discrete steps as individual subdomains emerge from the ribosome. The first step involves abrupt compaction of the N-terminal ATP binding subdomain (residues 389–500), which occurs on a time scale similar to or exceeding the predicted rate of translation. Because NBD1 has a very high contact order characterized by a large number of long-distance intrachain interactions, it is likely that the N-terminal subdomain provides a template or scaffold upon which the α-helical subdomain and α/β-core assemble. Finally, ΔF508 does not measurably influence N-terminal subdomain folding, indicating that the ΔF508 defect occurs during later folding of α-helical and/or α/β-core subdomains.

### CFTR domain–domain assembly

**Figure 4:**
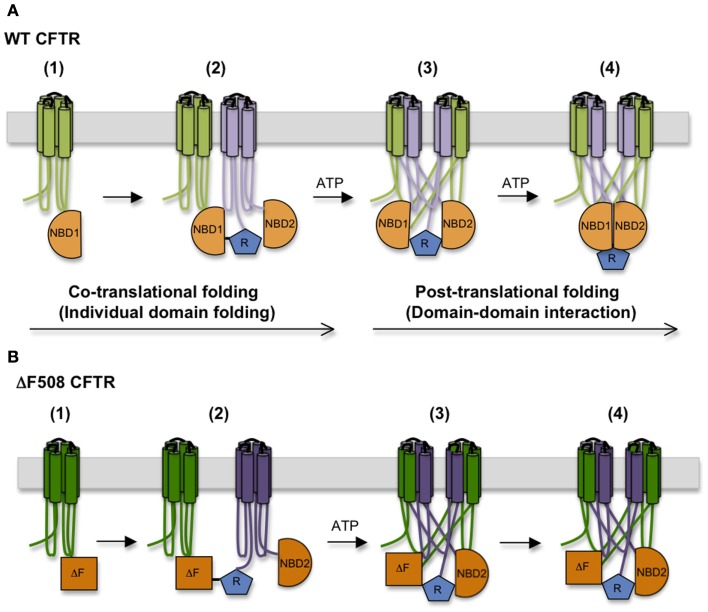
**Step-wise CFTR folding pathway**. **(A)** CFTR folding begins cotranslationally as individual domains are synthesized, and proceeds as domains assemble into a mature tertiary structure. **(B)** The ΔF508 mutation destabilizes NBD1 structure, interferes with the TMD1, TMD2, and NBD2 folding, and disturbs interactions between NBD1 and ICL4, compromising domain–domain assembly.

Based on the time required for CFTR to exit the ER, CFTR domain assembly takes ∼30–120 min. This suggests a hierarchical process in which domain folding begins cotranslationally and is followed by posttranslational formation of domain–domain contacts (Ostedgaard et al., 1997; Du et al., 2005; Cui et al., 2007; Figure 4). During this time CFTR undergoes at least two distinct folding events that require ATP. The first involves release of full length CFTR from a large biosynthetic complex that likely includes the RTC and cellular chaperones, and appears to coincide with CFTR integration (i.e., release) into the bilayer of the ER membrane (Meacham et al., 1999; Oberdorf et al., 2005). Both WT and ΔF508 CFTR undergo this step with equal efficiency (Oberdorf et al., 2005). As discussed above, delayed integration of TMDs may reflect the time required to establish the complex contacts within the domain swap structure. It is not known how ATP hydrolysis facilitates membrane integration, however, as no known translocon components hydrolyze ATP. The second maturation step involves conversion of CFTR from an immature, incompletely folded, ER-associated conformation (typically designated as Band B) to a properly folded, mature conformation that is competent to exit the ER and undergo Golgi processing into the Band C form (Lukacs et al., 1994). Interestingly, trapping CFTR in the ER with Brefeldin A results in accumulation of a stable, “mature” Band B form that is able to exit the ER upon Brefeldin A washout, indicating that the key folding step is distinct from Golgi processing. This folding transition also involves reorganization and/or release of cytosolic chaperones (Yang et al., 1993; Meacham et al., 1999) and results in a substantial change in CFTR structure as demonstrated by limited proteolysis (Zhang et al., 1998). Importantly, the ΔF508 mutation prevents this latter step.

While the precise details of CFTR maturation remain a mystery, WT and ΔF508 conformations differ in several important aspects. First, the complement of bound chaperones changes significantly; Hsp/c70 is released from WT CFTR prior to ER export, but remains bound to ΔF508 CFTR and may stimulate degradation (Yang et al., 1993; Matsumura et al., 2011). Second, the biological stability (as measured by half-life) of ΔF508 CFTR is more temperature sensitive than fully folded WT CFTR both in the ER and at the plasma membrane (Zhang et al., 1998). Structural differences between WT and mutant proteins can therefore be readily distinguished both by ER and peripheral quality control machinery (Okiyoneda et al., 2010). Third, channel activity of ΔF508 CFTR is more thermo-labile than WT and rapidly declines at physiological temperatures (37°C; Aleksandrov et al., 2010; Wang et al., 2011; Liu et al., 2012). Fourth, ΔF508 CFTR following maximal stimulation by PKA is less biologically stable than quiescent channels (Liu et al., 2012), indicating that features of the ΔF508 defect are mechanistically linked to conformational changes that take place during the gating cycle. Finally, different mechanisms of ΔF508 correction (e.g., low-temperature rescue, suppressor mutations, or small molecules) can be accomplished by a variety of structural changes that give rise to channels with different physical properties.

In addition to directly destabilizing NBD1 and weakening the interface between NBD1 and TMD2, limited proteolysis and cysteine crosslinking studies indicate that ΔF508 also causes conformational abnormalities in TMD1, TMD2, and NBD2 and misassembly of TMD1/TMD2 and NBD1/NBD2 interfaces (Du et al., 2005; Cui et al., 2007; Loo et al., 2008; Rosser et al., 2008; Du and Lukacs, 2009; He et al., 2010; Thibodeau et al., 2010). This high degree of cooperativity in CFTR domain folding is further supported by CF-related mutations in TMD1 and TMD2 that also reciprocally affect the conformation of other domains (Du and Lukacs, 2009).

## The Role of Chaperones in CFTR Folding

Because CFTR folding takes place in three different compartments, the ER lumen, the ER membrane, and the cytosol, CFTR interacts with several large cellular chaperone and co-chaperone networks (at least 31 components) at various stages of folding (Skach, 2006; Wang et al., 2006). Major chaperone families include cytosolic Hsp70, Hsp90, and their co-chaperones (Yang et al., 1993; Loo et al., 1998; Meacham et al., 1999; Younger et al., 2004; Grove et al., 2011), as well as ER lumenal lectins calnexin and possibly calreticulin (Pind et al., 1994; Harada et al., 2006).

**Figure 5:**
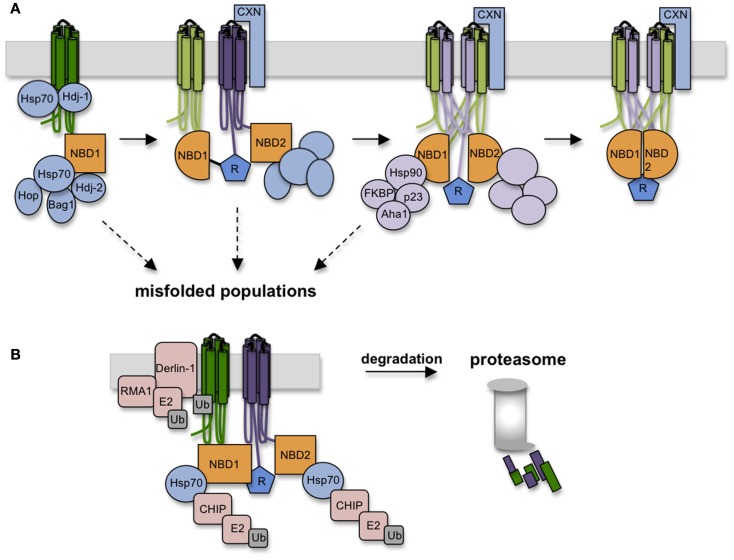
**Chaperones assist CFTR folding and target misfolded CFTR for degradation**. **(A)** As CFTR is synthesized, numerous chaperones and co-chaperones (some depicted here) decorate the nascent polypeptide on both lumenal and cytosolic sides of the ER membrane. Hsp70 and co-chaperones interact with NBD1, followed by calnexin association with TMD2. Hsp70-Hop interactions recruits Hsp90 complexes which likely aid domain assembly in conjunction with calnexin. **(B)** Failure to achieve productive folding at any step in the folding pathway is detected by persistent binding of Hsp70, which serves to recruit E3 ligases (i.e., RMA1 and CHIP) that ubiquitinate CFTR and target it to the 26S proteasome.

Cytosolic chaperone interactions begin cotranslationally during synthesis as Hsp/c70 binds and presumably shields extended hydrophobic regions of the nascent chain to prevent aggregation (Yang et al., 1993; Meacham et al., 1999; Oberdorf et al., 2005; Kampinga and Craig, 2010). Hsp/c70 binds substrate in the ATP-bound state, and binding is stabilized by ATP hydrolysis, which is stimulated by DnaJ (Hsp40) cofactors. Substrate is released upon nucleotide exchange, which can be either spontaneous, or stimulated by nucleotide exchange factors (NEFs) such as Bag-1 and HspBP1. While details of Hsp/c70-CFTR interactions are far from complete, peptide binding studies have identified potential binding sites in NBD1, and have shown that Hsc70 decreases ΔF508 NBD1 aggregation *in vitro* possibly by reducing off-pathway folding events (Strickland et al., 1997; Figure 5). In cells, both Hsc70 and Hdj-2 interact with CFTR after the NBD1 synthesis but are released in the presence of the R-domain (Meacham et al., 1999). In addition, Hsp70 and Hdj-1 coexpression stabilizes WT CFTR *in vivo* (Farinha et al., 2002), pointing out the critical role of Hsp70 in CFTR NBD1 folding in the cytosol. Later stages of TMD2 folding and TMD1 and TMD2 assembly appear to require calnexin (Rosser et al., 2008) which likely binds TMD2 via N-linked glycans attached to ECL4. This interaction may stabilize TMD2 and/or assist in orienting TMs during domain swapping. Taken together, these findings suggest that CFTR utilizes a carefully orchestrated complement of chaperones at numerous sequential and interdependent folding steps (Figure 5).

Paradoxically, chaperones that facilitate CFTR folding also play a direct role in degradation. The best understood example is Hsp70, which resides squarely at the intersection of folding and quality control. Pro-folding activities of Hsp/c70 are mediated through its N-terminal ATPase domain, which controls affinity of the central peptide binding cleft. The C-terminus of Hsc70, however, contains a tetratricopeptide binding motif that interacts with at least one E3 ubiquitin ligase, CHIP, that functions in concert with the E2 ubiquitin conjugating enzyme, UbcH5 (Meacham et al., 2001; Younger et al., 2004). While other E3 ligases are also implicated in CFTR ubiquitination (e.g., Nedd4-2, RMA1, and gp78; Younger et al., 2006; Morito et al., 2008; Caohuy et al., 2009; Grove et al., 2011), Hsc70-CHIP seems to play a major role in recognizing cytosolic structural perturbations caused by ΔF508 (Meacham et al., 2001; Younger et al., 2004).

A non-trivial question therefore is how Hsp/c70 carries out two diametrically opposed actions, on the one hand protecting proteins from aggregation and facilitating folding, while on the other identifying terminally misfolded proteins and targeting them for degradation. An important clue was recently provided by Matsumura et al. (2011) who used a C-terminal fragment of Bag-1 to stimulate Hsc70 nucleotide exchange (Höhfeld and Jentsch, 1997; Takayama et al., 1997). Addition of cBag during CFTR translation slightly increased degradation, consistent with predictions that Hsp70-client interactions stimulate *de novo* folding (Meacham et al., 1999; Younger et al., 2004; Grove et al., 2011), whereas similar levels of cBag completely blocked CFTR degradation, consistent with studies in yeast (Zhang et al., 2001) and mammalian cells (Farinha et al., 2002). Kinetic analysis revealed that shortening the time required for CFTR-Hsc70 dissociation from roughly 3 min to less than 1 min resulted in a marked decrease in CFTR ubiquitination and degradation. Thus, the timing of the Hsp70 binding cycle, rather than binding *per se*, appears to be a critical decision point in the degradation process.

Hsp/c70 also recruits Hsp90 complexes through the intermediate linker protein p60 (Hop; Frydman and Höhfeld, 1997). In contrast to Hsp/c70, Hsp90 appears to primarily enhance CFTR folding (Loo et al., 1998). During its binding cycle, conformational shifts in Hsp90’s client binding interface likely induce structural changes in substrate that mediate conversion from immature to mature conformations. Hsp90-client binding is also regulated by a variety of co-chaperones that include p23, cyclophilins (i.e., FKBPs), and Aha1, each of which associates with CFTR in cells (Wang et al., 2006; Hutt et al., 2012). While it is not yet known precisely how Hsp90 affects CFTR folding, overexpression of the co-chaperone Aha1, which stimulates Hsp90 ATPase activity and client release, decreases ΔF508 CFTR stability, and Aha1 knockdown enhances ΔF508 processing. Thus, stabilization of CFTR Hsp90 binding increases the dwell time of CFTR in the Hsp90 complex, which may overcome a kinetic bock in CFTR folding (Qu et al., 1997; Skach, 2006; Koulov et al., 2010). A recent study has also shown that an additional Hsp90 co-chaperone, a peptidylprolyl isomerase, FKBP8, interacts with and stabilizes both WT and ΔF508 CFTR in the ER via a mechanism that requires prolyl-isomerase activity (Hutt et al., 2012). Thus the Hsp90 axis is a potentially attractive target for CFTR correction.

## Summary

In summary, research in the past decade has revealed much about how CFTR folds and misfolds in cells. Membrane insertion and tertiary folding of cytosolic domains begin cotranslationally during CFTR synthesis, whereas posttranslational folding involves assembly of TM helical bundles that provide critical domain–domain contacts needed to form a physiologically stable structure. While the details of this process require further refinement at the molecular level, the model that emerges from these studies provides a useful framework to understand the key folding defect(s) caused by ΔF508 in the majority of CF patients. Within NBD1 itself, removal of Phe508 decreases folding efficiency and renders the domain susceptible to unfolding, denaturation, and aggregation at physiologic temperatures, possibly as a direct result of destabilizing the α-helical subdomain. Absence of Phe508 also disrupts the interaction between NBD1 and ICL4 (within TMD2), which distorts TMD structure and interferes with channel gating. Defects in NBD1 and the NBD1–ICL4 interface are both recognized by quality control machinery, and correction of both is necessary and sufficient to restore trafficking and function to near WT levels. Importantly, partial correction of ΔF508 CFTR folding can be achieved by a variety of means: cis-acting suppressor mutations, manipulation of the proteostatic network, or small molecule correctors. Moreover, combinations of these maneuvers are now able to achieve near WT levels of surface expression and function. Thus, it is increasingly attractive to target the next generation of CFTR small molecule correctors to specific defects that will optimize synergy in correction mechanisms. While the most precise targets reside within the CFTR molecule itself, i.e., NBD1 and the NBD1–TMD2 interface, it is also possible that other clinically beneficial targets will be developed in the years to come, which will undoubtedly be driven by increasing resolution of the folding problem.

## Conflict of Interest Statement

The authors declare that the research was conducted in the absence of any commercial or financial relationships that could be construed as a potential conflict of interest.

## Abbreviations

CF, cystic fibrosis; CFTR, cystic fibrosis transmembrane conductance regulator; ER, endoplasmic reticulum; NBD, nucleotide binding domain; PCC, protein conducting channel; RNC, ribosome nascent chain complex; RTC, ribosome translocon complex; TM, transmembrane segment.
